# etymologia: *Coxiella* [kok′′ se-el′ ә] *burnetii*

**DOI:** 10.3201/eid1410.080000

**Published:** 2008-10

Etiologic agent of Q fever, named after American bacteriologist Herald Rea Cox and Australian physician Frank MacFarlane Burnet, who both independently isolated the bacterium in the 1930s. *C. burnetii* belongs in the family *Coxiellaceae*, which consists of gram-negative rods without flagella or a capsule. The bacteria occur in ticks and various vertebrates, including humans. 

